# Influence of chlorhexidine dentin disinfection on universal adhesive performance: Interfacial adaptation and bond strength assessments

**DOI:** 10.1371/journal.pone.0315036

**Published:** 2024-12-31

**Authors:** Alaa Turkistani, Helal M. Sonbul, Mai Almarzouki

**Affiliations:** Departement of Restorative Dentistry, Faculty of Dentistry, King Abdulaziz University, Jeddah, Saudi Arabia; University of New Hampshire, UNITED STATES OF AMERICA

## Abstract

**Purpose:**

This study aimed to investigate the effect of chlorhexidine (CHX) cavity disinfectant on interfacial microleakage and micro-tensile bond strength (μTBS) of a universal adhesive bonded to dentin in both self-etch (SE) and etch-and-rinse (ER) modes.

**Methods:**

Class I cavities were prepared in the coronal dentin of extracted human teeth and assigned to two etching modes (SE or ER), then subdivided by disinfection with or without CHX (n = 5). Cavities were restored using Single Bond Universal Adhesive and Filtek Z350 XT composite. After 10,000 thermal cycles, interfacial microleakage was assessed on serial B-scans obtained for each specimen using cross-polarization optical coherence tomography. For μTBS testing, resin composite was bonded to dentin discs, sectioned into beams, and subjected to tensile load until failure using a universal testing machine. Failure modes in fractured beams were analyzed under a stereomicroscope and categorized as adhesive, cohesive, or mixed. ANOVA with Bonferroni post-hoc at a significance level of 0.05 was used to compare the groups.

**Results:**

Microleakage was not significantly influenced by the etching mode or CHX disinfection (p-value = 0.068). For μTBS, the ER group exhibited the highest values, and CHX disinfection did not significantly alter these results (p-value = 1.000). In contrast, the SE-CHX group displayed significantly lower μTBS than the ER, ER-CHX, and SE groups (p-values of <0.001, <0.001 and 0.012, respectively). ER mode primarily resulted in adhesive failures, regardless of CHX. SE group exhibited both adhesive and cohesive failures, while SE-CHX mostly showed adhesive failures.

**Conclusion:**

The use of CHX disinfectant influences the bonding performance of universal adhesive differently depending on the application mode. Specifically, in the SE mode, CHX adversely affects bond strength to dentin. This suggests that when using universal adhesives in SE mode, clinicians should carefully consider the use of CHX disinfectants, as they may interfere with the adhesive’s effectiveness.

## Introduction

Achieving a durable bond between resin composite restorations and tooth structure is a key goal in adhesive dentistry. Despite advancements in adhesive systems, bonding to dentin remains a challenge. Unlike enamel, dentin is a complex, fluid-filled structure with hydroxyapatite crystals embedded in a collagen matrix. For effective bonding, a stable hybrid layer of demineralized collagen fully impregnated by polymerized resin at the adhesive-dentin interface must be achieved [1, 2]. Inadequate integrity of this adhesive interface can lead to microleakage, ultimately compromising the longevity of the restoration [1].

A primary cause of bond failure is the degradation of exposed collagen fibrils by cathepsins and matrix metalloproteinases (MMPs), particularly MMP-2, -8, and -9. These enzymes are activated following demineralization and degrade the hybrid layer, thereby increasing the risk of microleakage and bond failure [3–5]. In addition to enzymatic degradation, bacterial infiltration poses another significant risk to restoration integrity. Histological evidence indicates that cariogenic bacteria can persist within dentinal tubules, the smear layer, and at the dentin-enamel junction even after caries excavation and cavity preparation [6]. While residual bacteria do not always cause caries progression, especially when materials effectively seal dentinal surfaces and inhibit bacterial growth [7], inadequate sealing in the presence of residual bacteria can lead to significant complications [8]. Furthermore, compromised margins may allow bacterial infiltration, promoting biofilm formation and secondary caries [9].

To mitigate these risks, the use of adhesives with inherent antibacterial properties or antibacterial agents, such as chlorhexidine (CHX), for cavity disinfection has been suggested as a valuable clinical strategy [10, 11]. CHX is a broad-spectrum antimicrobial agent that can also inhibit the activation of MMPs, thus preserving the dentin matrix and potentially improving bond integrity [3, 5, 12, 13]. However, the overall impact of CHX on bonding performance remain subjects of debate. While some studies have demonstrated enhanced bond strength with CHX, others report either no improvement or an increase in microleakage [14–19].

Nowadays, the use of universal adhesives is increasingly popular among clinicians due to their simplified bonding process, shorter application time, and versatility in bonding to various substrates. However, their complex formulation may increase microleakage and potentially stimulate MMPs activity, which raises concerns about the higher failure rates when used in conjunction with CHX [20–22]. Traditional methods such as dye penetration and microscopic evaluation have long been used to assess microleakage. Alternatively, advanced imaging technologies like optical coherence tomography (OCT) now provide more sensitive, real-time, non-destructive assessment, offering significant advantages in evaluating restoration integrity [23, 24].

The current in vitro study tested the effect of disinfecting dentin with CHX on the microleakage and bond strength of resin composite restorations bonded to dentin with a universal adhesive in self-etch and etch-and-rinse modes. The null hypothesis tested was that disinfecting dentin with CHX does not significantly affect interfacial microleakage or the bond strength of a universal adhesive to dentin in either self-etch or etch-and-rinse modes.

## Materials and methods

### Sample size and power analysis

The effect size (Cohen’s f) was calculated to be approximately 3.58, which is considered large. A statistical software (G*Power; Heinrich-Heine-Universität Düsseldorf, Düsseldorf, Germany) was used to determine that a large effect size (0.9) required a total sample size of 20 (5 samples per group, with four groups in total) to achieve a statistical power of 0.8 with a significance level of α = 0.05. In each group, 5 teeth were used, and 10 serial cross-sectional images were obtained per tooth and averaged to improve measurement reliability.

### Specimens preparation

All procedures in this study were conducted in accordance with the Declaration of Helsinki. The present study was ethically approved by “King Abdulaziz University Ethical Committee” on 03/04/2019 under protocol number 069-03-19. The committee deemed the project exempt from full review. Given the minimal risk involved, the ethics committee waived the requirement for informed written consent.

**Fig 1:**
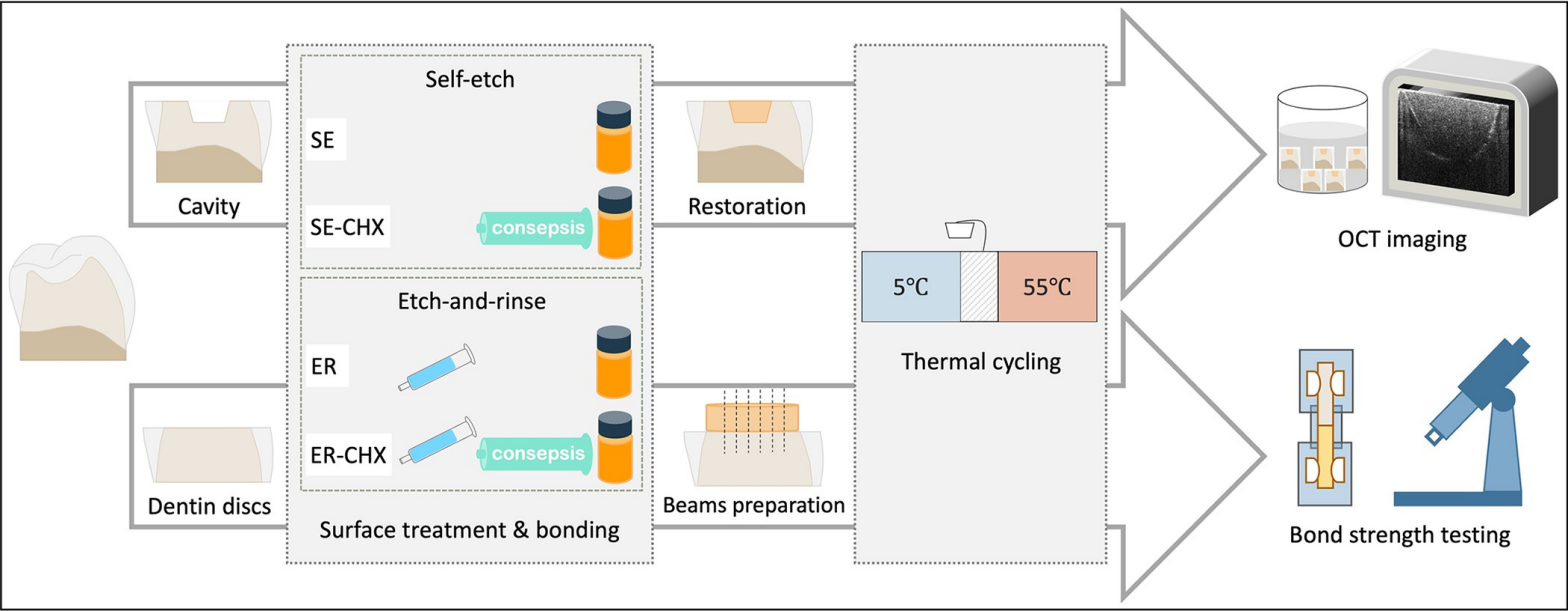
Schematic illustration of the study design. This figure is for illustrative purposes only; the individual elements are similar but not identical to the originals.

A schematic drawing of study design is presented in Fig 1. A total of twenty extracted human molar and premolar teeth without evidence of caries or crack were collected and preserved in saline. Patients were informed that their extracted teeth would be used for scientific research, and all provided verbal consent. Using a model trimmer (MT 10; Ray Foster, Huntington beach, CA, USA), the cusps were trimmed at a perpendicular angle to the long axis of the tooth to expose coronal dentin and roots were separated using a diamond discs (Meisinger Diamond Discs; Meisinger, Centennial, CO, USA). Standardized cylindrical class I cavities with 4-mm width and 2-mm depth were prepared using a coarse diamond bur (Meisinger Diamond Instruments 811G; Meisinger, Centennial, CO, USA) coupled to a water-cooled highspeed handpiece, followed by fine diamond bur for finishing (Meisinger Diamond Instruments 881F; Meisinger, Centennial, CO, USA). Burs of each type were changed every five preparations. Then, the teeth were randomly divided into two main groups (n = 10) based on the etching mode; self-etch (SE) or etch-and-rinse (ER). In the ER group, phosphoric acid etchant (Scotchbond Universal Etchant; 3M ESPE, St Paul, MN, USA) was applied for 15 s, rinsed thoroughly for 10 s and dried with a cotton pellet. Each group was further divided into two subgroups based on CHX treatment (n = 5); one subgroup without CHX and the other with CHX. In CHX treated subgroups, 2% Chlorhexidine Gluconate disinfecting solution (Consepsis; Ultradent Products, South Jordan, UT, USA) was applied for 60 s and gently air-dried. After that, cavities in all groups were restored with a universal adhesive (Single Bond Universal adhesive; 3M ESPE, St. Paul, MN, USA) and a single increment of universal nano-composite (Filtek Z350 XT Universal Restorative; 3M ESPE, St. Paul, MN, USA) using an LED curing unit (Bluephase N cordless light cure; Ivoclar Vivadent, Schaan, Liechtenstein). The composition and application instructions of the used materials are shown in Table 1. After 24 h storage in relative humidity, specimens underwent thermal cycling. The cycling involved 10,000 cycles between 5°C and 55°C, with 30 s dwell time at each temperature and a 10 s transfer time between baths (Thermocycler THE-1100; SD Mechatronik, Feldkirchen-Westerham, Germany).

**Table 1:** Composition and application instructions of the materials used in the study.

Material	Composition	Application instructions
Manufacturer		
Consepsis	2% Chlorohexidine Gluconate disinfecting solution	Rub the solution thoroughly and leave for 60 s, air dry gently
Ultradent (South Jordan, UT, USA)		
Single Bond Universal Adhesive	MDP Phosphate monomer, vitrebond copolymer, HEMA, dimethacrylate resins, filler, silane, initiators, ethanol, water	Apply the adhesive and rub it for 20 s, gentle air dry for 5 s, light cure for 10 s
3M ESPE (St. Paul, MN, USA)		
Filtek Z350 XT Universal Restorative	Bis-GMA, bis-EMA, UDMA, TEGDMA, zirconia/silica fillers	Apply in layers up to 2 mm in thickness and light cure for 20 s
3M ESPE (St. Paul, MN, USA)		

Abbreviations: MDP, 10-methacryloyloxydecyl dihydrogen phosphate; HEMA, 2-hydroxyethyl methacrylate; Bis-GMA, bisphenol A diglycidyl methacrylate; bis-EMA, bisphenol A diglycidyl methacrylate ethoxylated; UDMA, urethane dimethacrylate; TEGDMA, triethylene glycol dimethacrylate.

After thermal cycling, specimens were covered with varnish (Nail Enamel; Revlon, New York, NY, USA), leaving a 1 mm space around the restoration margins. Subsequently, they were stored in a dark environment within an ammoniacal silver nitrate solution. This solution was prepared by dissolving silver nitrate (Sigma-Aldrich Silver Nitrate; Sigma-Aldrich, St. Louis, MO, USA) in distilled water and titrating with ammonium hydroxide (Sigma-Aldrich Ammonium Hydroxide Solution; Sigma-Aldrich, St. Louis, MO, USA). After 24 h, the specimens were removed, thoroughly rinsed under running water, and exposed to a photo-developing solution (Kodak GBX fixer and replenishers; Kodak, Rochester, NY, USA) for 8 h under fluorescent light.

### Interfacial adaptation

In this study, cross-polarization OCT system (CP-OCT IVS-300; Santec, Komaki, Japan) was used to evaluate interfacial microleakge. The wavelength ranged from 1300 to 1360, with a central wavelength of 1330 nm. The system has a sensitivity of 95 dB and with optical axial resolution of 12 μm in depth and lateral resolution of 30 μm [24].

During the scan, the specimen was placed on a micrometer platform and scanning beam was directed perpendicularly at 90° onto the occlusal surface of the restoration using a handheld probe at a fixed distance. In depth, 10 serial B-scans along the x, y axes were obtained from each specimen. To quantify the microleakage, primary data were imported to image analysis software (ImageJ; National Institutes of Health, Bethesda, MD, USA), and a median filter was applied to decrease background noise. A rectangle selection was drawn around the cavity floor, defining the restoration interface. Interfacial microleakage in OCT image is indicated by pixels with increased signal intensity compared to adjacent pixels at the interfacial zone. A binary image of the selection was then obtained, and total width of pixels with high signal intensity was divided by cavity floor width to calculate the microleakage percentage.

### Microtensile Bond Strength (μTBS)

To measure the bond strength, dentin discs were obtained from extracted human teeth. The surface of each disc was prepared to standardize the smear layer using sandpaper (600 Grit Silicon Carbide Waterproof Sandpaper; ACE Hardware, Oak Brook, IL, USA) under copious water for 60 s. Resin composite was then bonded to the dentin discs using the same procedure as for preparing the microleakage specimens. After 24 h of storage in humid condition, the specimens were sectioned into beams using a diamond saw (IsoMet Low Speed Precision Cutter; Buehler, Lake Bluff, IL, USA), resulting in a total of ten beams per group (n = 10). The beams were subjected to 10,000 thermocycles. Next, the beams were glued to customized jigs and stressed using a universal testing machine (Multitest 2.5-i; Mecmesin, Sterling, VA, USA) at a crosshead speed of 1 mm/min until failure. The μTBS was determined by dividing the fracture force by the cross-sectional area of the beam. Fractured halves of the beams were observed under stereomicroscope (RaySmart Digital stereomicroscope; Shenzhen RaySmart Technology, Shenzhen, China) to evaluate and categorize the failure mode as either adhesive, cohesive, or mixed failure.

### Statistical analysis

Descriptive statistics (mean and standard deviation) were presented for each group. Normality of microleakage percentage and μTBS were assessed by Shapiro-Wilk test, and no deviations from normality were observed. Bartlett’s test for equal variances indicated homogeneity across the groups, ensuring the appropriate use of ANOVA. ANOVA was employed to compare the mean of microleakage percentage and μTBS among the groups, followed by multiple comparisons using Bonferroni post-hoc test. Statistical significance was set at a p-value ≤ 0.05. Data analysis was performed using statistical software (Stata version 12.1; Stata, College Station, TX, USA).

## Results

**Fig 2:**
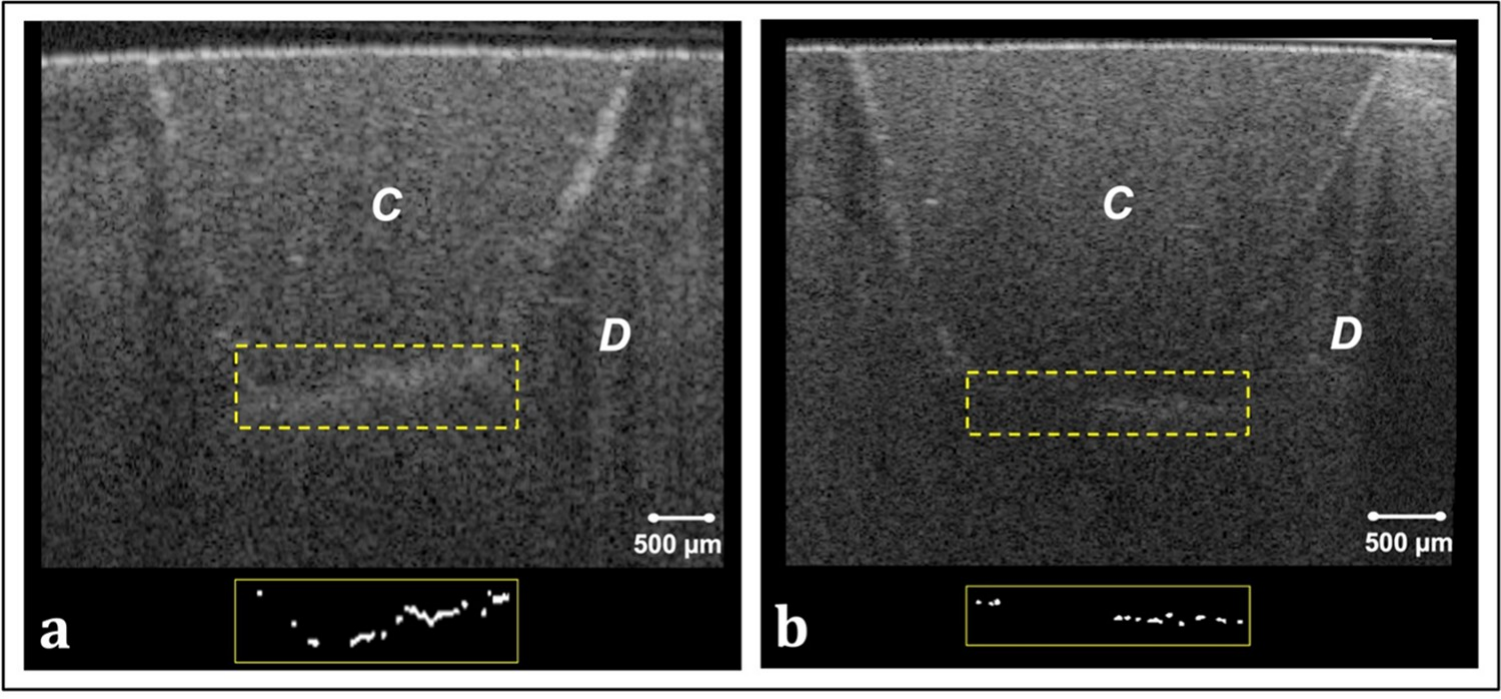
Representative cross-sectional images of selected specimens from ER and ER-CHX groups, along with corresponding binary images after binarization of the interfacial area. (a) B-scan of a specimen from ER group, showing increased signal intensity and diffuse reflections at the cavity floor. Bright pixels in the corresponding binary image highlight interfacial microleakage. (b) B-scan of a specimen from ER-CHX group, similarly revealing interfacial microleakage at the cavity floor, with bright pixels in the binary image indicating areas of leakage. C: resin composite; D: dentin.

**Fig 3:**
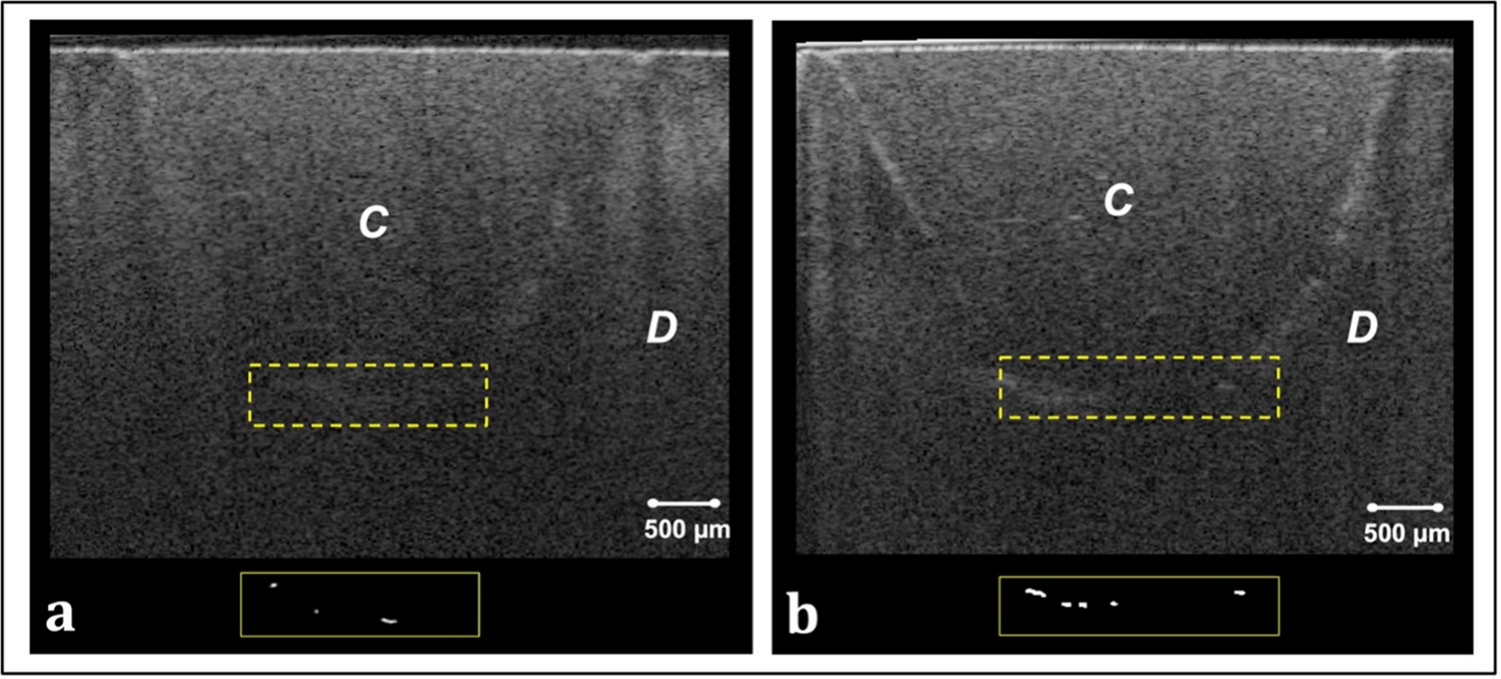
Representative CP-OCT images obtained from specimens of SE and SE-CHX groups, along with corresponding binary images of the resin composite-dentin interface at the cavity floor. (a) B-scan of a specimen from SE group, showing a few scattered areas of increased signal intensity at the cavity floor. The binary image highlights these areas as isolated bright pixels along the interfacial region. (b) B-scan and binary image of a selected interface from SE-CHX specimen, showing slightly more microleakage than the SE specimen. C: resin composite; D: dentin.

Representative CP-OCT images and corresponding binary images of the bonded interfaces are shown in Figs 2 and 3. Interfacial microleakage appearing as increased signal intensity and clusters of bright pixels at the cavity floor in CP-OCT images was frequently observed in ER and ER-CHX groups (Fig 2). In SE group, however, some B-scans demonstrated low signal intensity at the floor of the cavity which represents adequate adaptation. Occasionally, scattered bright clusters were observed at the bonded interface of SE group (Fig 3(A)). This occurrence was more pronounced with CHX treatment (Fig 3(B)).

Table 2 illustrates the mean and standard deviation of microleakage percentage for the four groups: ER, ER-CHX, SE, SE-CHX. The mean [± SD] of microleakage percentage value ranged from 27.3 [±1.9] to 34.3 [±5.6], with the lowest value observed in SE and the highest in ER group. ANOVA results demonstrated that there was no statistically significant difference between overall mean microleakage percentage values of the test groups (p-value = 0.068).

**Table 2:** Mean and standard deviation of the microleakage percentage at the cavity floor of the tested groups.

Variable	Mean	Std. Dev.	p-value
Etch-and-rinse (ER)	34.3	5.6	0.068
Etch-and-rinse-chlorhexidine (ER-CHX)	33.5	4.7	
Self-etch (SE)	27.3	1.9	
Self-etch-chlorhexidine (SE-CHX)	30.1	4.0	

μTBS differed among the groups, as shown in Table 3. The ER group displayed the highest [± SD] mean value, 27.5 [±2.9]. ANOVA result showed that when CHX was used after acid etching (ER-CHX), the μTBS value did not change significantly to that of ER or SE groups (p-value = 1.000 and p-value = 0.714, respectively). Similarly, the SE group showed no significant change in mean compared to the ER group (p-value = 0.416). When CHX was used with SE mode, the μTBS value significantly decreased compared to the SE (p-value = 0.012), ER-CHX (p-value <0.001), and ER groups (p-value <0.001).

**Table 3:** Comparison of bond strength (μTBS, MPa) with four test groups.

Variable	Mean	Std. Dev.		p-value		Multiple comparisons	Failures A/C/M
Etch-and-rinse (ER)	27.5		2.9		<0.001	ER vs ER-CHX	9/1/0
						ER vs. SE	
						ER vs. SE-CHX*	
Etch-and-rinse-chlorhexidine (ER-CHX)	26.7		4.9			ER-CHX vs SE	9/1/0
						ER-CHX vs SE-CHX*	
Self-etch (SE)	22.5		5.2			SE vs. SE-CHX*	6/4/0
Self-etch-chlorhexidine (SE-CHX)	13.1		2.3				9/0/1

*Multiple comparisons associations significant at 0.05 level: ER vs SE-CHX, ER-CHX vs SE-CHX, SE vs SE-CHX. A: adhesive failure; C: cohesive failure; M: mixed adhesive and cohesive failure.

^Bonferroni post-hoc for multiple comparisons

Microscopic evaluation of the beams following μTBS testing revealed that specimens bonded using the etch-and-rinse mode predominantly exhibited failures that occurred at the adhesive interface, even when CHX was used. In the self-etch mode with CHX disinfection, adhesive failures were also the most common. However, in the self-etch mode without CHX, a combination of adhesive and cohesive failures was observed (Table 3).

## Discussion

The durability of dental restorations can be anticipated based on their capacity to adhere effectively to dental structures, often assessed through bond strength evaluation. Additionally, microleakage testing could reveal potential gaps or infiltration at the adhesive-tooth interface, providing crucial insights into the restoration’s sealing quality. High bond strength, alongside with minimal microleakage, contribute to restoration’s long-term success and durability in the challenging oral environment [25]. The current in vitro study tested the bonding performance of Single Bond Universal adhesive applied in self-etch and etch-and-rinse modes with or without disinfecting the cavity using CHX. The null hypothesis for microleakage was not rejected, as CHX disinfection did not significantly affect microleakage, regardless of the etching mode. For μTBS, the null hypothesis was not rejected in the etch-and-rinse mode, but it was rejected in the self-etch mode, indicating a significant effect of CHX on bond strength in the latter.

In this study, a 2% CHX concentration was selected and applied following acid etching without subsequent rinsing. This specific protocol was chosen based on manufacturer instructions and supported by findings from relevant literature, where similar approaches have been recommended and validated [26]. CP-OCT was employed in the study to identify the interfacial microleakage in composite restorations. OCT is recognized as a nondestructive diagnostic imaging tool that utilizes a wideband light source to generate high-resolution images [27]. Previous studies have demonstrated OCT’s notable capability to detect interfacial gaps and microleakage in resin composite restorations, offering valuable insights into the integrity of the bonding interface without the need to cut the specimen [28]. Interfacial gaps can be detected using OCT due to the contrast in reflection between the imaged structures and the air or water presumed to fill the gap [29]. A previous study demonstrated that combining a contrast agent with CP-OCT allowed for more accurate detection of interfacial gaps in tested samples [24]. Thus, silver nitrate was utilized as a contrast agent in this study. The diffusion of silver granules into the microgaps enabled enhanced light reflection and improved contrast of the observed microgaps in CP-OCT cross-sections.

The results of this study showed that microleakage was not significantly influenced by either the etching mode or CHX disinfection. Thus, null hypothesis for microleakage was not rejected. Single bond universal adhesive used in this study is considered an ultra-mild adhesive (pH = 2.7) that contains the functional acidic monomer 10-methacryloyloxydecyl dihydrogen phosphate (10-MDP) and polyalkenoic acid co-polymer. OCT images of etch-and-rinse application mode revealed increased bright clusters of interfacial microleakage, that extended across the entire cavity floor in some cross-sections. The increased microleakage can be attributed to the aggressive nature of phosphoric acid, which removes the smear layer and leads to deeper demineralization [30]. This demineralization, while inherent to the etching process, can be exacerbated by improper technique, exposing the collagen matrix to hydrolytic and enzymatic degradation over time [31]. Inadequate moisture control can also lead to water entrapment, interfering with resin polymerization and causing incomplete monomer conversion, hydrolytic degradation, and nanoleakage. Additionally, the depth of demineralization and entrapped water may result in inconsistent resin infiltration, further compromising the adhesive interface [32]. Moreover, the reduction in hydroxyapatite, essential for bonding with functional monomers like 10-MDP, diminishes the adhesive’s chemical bonding potential. Conversely, the self-etch technique partially etches dentin, leaving some hydroxyapatite around the collagen matrix. Therefore, 10-MDP is capable of forming ionic bonds with the residual hydroxyapatite, creating a stable, self-assembled 10-MDP-Ca nano-layer at the interface, which reduce the likelihood of interfacial gaps and contribute to bond stability [22].

Certain studies concluded that CHX had no adverse effect on interfacial sealing of etch and rinse adhesives [14, 33]. These findings align with the outcomes of this study, showing no effect on cavity floor microleakage of ER-CHX compared to ER group. On the other hand, research on CHX’s influence on microleakage with self-etch adhesive systems has yielded conflicting results. While some studies found no significant changes [14], others reported an increase in microleakage when CHX was used with self-etching single-bottle adhesives, in agreement with the current findings [18]. OCT images of self-etch bonded interface showed slightly more microleakage with CHX application, though the difference was not statistically significant. Furthermore, a recent study evaluated marginal gaps in composite restorations bonded using self-etch and etch-and- rinse bonding strategies and concluded that marginal gap sizes in the CHX groups were significantly larger compared to the non-CHX groups [16].

In this study, results of the μTBS test showed higher bond strength when the adhesive was used in etch-and-rinse mode. This can be explained by the thicker hybrid layer and rougher dentin surface achieved by phosphoric acid etching, as supported by previous research [21, 30]. Phosphoric acid etching also dissolves intratubular mineral deposits, facilitating deeper resin tag formation compared to the self-etch mode. While resin tag formation plays a role in adhesion, it has been suggested that chemical bonding to hydroxyapatite is the primary mechanism, and the length of the resin tags should not have any impact on the bond strength [19].

When CHX was applied after phosphoric acid etchant, the μTBS was not adversely affected or impaired. This outcome supports the positive impact of CHX on the bonding interface by preserving collagen matrix integrity and inhibiting collagenolytic enzymes [4, 5, 34]. A recent zymography study demonstrated that CHX persists within the hybrid layer and maintains its effect after 10 years of immersion in artificial saliva [34]. Additionally, CHX appears to possess the capability to eliminate loose smear debris and enhance the free energy of the surface etched with phosphoric acid, thereby improving the wetting ability of primers [13]. Its use as an additional primer has been shown to significantly reduce bond strength loss and nanoleakage in acid-etched dentin over a 2-year aging period [35]. Furthermore, a study reported that the use of CHX with etch-and-rinse adhesives preserved the bond strength to both sound and eroded dentin [36].

Conversely, the use of CHX in conjunction with the self-etch mode significantly decreased the bond strength, which require partial rejection of the null hypothesis. This finding corroborates with the results of the previous studies [12, 34, 35]. CHX may precipitate on the dentin surface, interfering with acid conditioning and resin infiltration, thus hindering hybrid layer formation. A previous study suggested that dissociated CHX cations could interact with phosphate groups and calcium in hydroxyapatite. The residual cations following this interaction could potentially create bonds with phosphate anions of the 10-MDP functional monomer when the adhesives is applied to CHX-treated surfaces. This effect might compromise the bonding capability of 10-MDP with calcium of hydroxyapatite, leading to a reduction in bond strength to dentin [37]. Previous investigations have noted that the hybrid layer formed in dentin treated with CHX is manifested by reduced thickness, less consistency, and a diminished number of resin plugs [16]. Failure mode analysis supports these findings, as SE group specimens exhibited a mix of adhesive and cohesive failures, whereas SE-CHX specimens predominantly showed adhesive failures. This shift towards adhesive failure indicates weakening of the interface, further supporting the negative impact of CHX on bond strength in the self-etch mode.

In this study, several strengths contribute to the significance of the findings. The evaluation of CHX’s effect on bonding performance in both self-etch and etch-and-rinse modes provides a comprehensive view for diverse clinical practices. Additionally, the use of thermal cycling to simulate intraoral aging enhances the study’s clinical relevance by mimicking the thermal stresses restorations undergo in the oral environment, offering insights into bond durability. Furthermore, the application of OCT allowed for non-destructive, high-resolution assessment of microleakage, improving the accuracy of the results.

However, this study also has limitations. Only one adhesive was evaluated, which may limit the generalizability of the results. Future studies should assess different types of adhesives with varying functional monomers to better understand how CHX affects a wider range of materials. Additionally, the adhesive interface was not visualized using advanced techniques such as scanning electron microscopy (SEM), which could have provided more detailed insights into the interface structure of the experimental groups.

## Conclusion

The performance of universal adhesives can be influenced by the interaction between cavity disinfectants and the universal adhesive application mode. The findings of this study suggest that CHX can be applied with the etch-and-rinse mode without significantly compromising the adhesive effectiveness. However, caution is recommended when using CHX with the self-etch mode, as it may negatively impact the bond strength.

## Supporting information

S1 Dataset Dataset for experimental groups. (XLSX)
